# Prebiotics, Probiotics, Synbiotics, Paraprobiotics and Postbiotic Compounds in IBD

**DOI:** 10.3390/biom11121903

**Published:** 2021-12-18

**Authors:** Adrian Martyniak, Aleksandra Medyńska-Przęczek, Andrzej Wędrychowicz, Szymon Skoczeń, Przemysław J. Tomasik

**Affiliations:** 1 Department of Clinical Biochemistry, Pediatric Institute, Faculty of Medicine, Jagiellonian University Medical College, 30-663 Krakow, Poland; adrian.martyniak@uj.edu.pl; 2 Department of Paediatrics, Gastroenterology and Nutrition, Pediatric Institute, Faculty of Medicine, Jagiellonian University Medical College, 30-663 Krakow, Poland; aleksandra.medynska@gmail.com (A.M.-P.); andrzej.wedrychowicz@uj.edu.pl (A.W.); 3 Department of Pediatric Oncology and Hematology, Faculty of Medicine, Jagiellonian University Medical College, 30-663 Krakow, Poland; szymon.skoczen@uj.edu.pl

**Keywords:** ulcerative colitis, Crohn’s disease, inflammatory bowel disease, intestinal microbiome, short chain fatty acids, dietary fibers

## Abstract

The increasing incidence of inflammatory bowel diseases (IBD) and the increasing severity of the course of these diseases create the need for developing new methods of therapy. The gut microbiome is extensively studied as a factor influencing the development and course of IBD. The composition of intestinal microbiota can be relatively easily modified by diet (i.e., prebiotics, mainly dietary fibers) and bacterial supplementation using beneficial bacteria strains called probiotics. Additionally, the effects of the improved microbiome could be enhanced or gained by using paraprobiotics (non-viable, inactivated bacteria or their components) and/or postbiotics (products of bacterial metabolism or equal synthetic products that beneficially modulate immunological response and inflammation). This study summarizes the recent works on prebiotics, probiotics, synbiotics (products merging pre- and probiotics), paraprobiotics and postbiotics in IBD.

## 1. Introduction

The human intestinal microbiome consists of over 1000 species of bacteria and other microorganisms. The total number of these may exceed the number of host cells [1]. The intestinal microflora, also called the microbiome, has many functions. Its primary function is to support the digestive system. In addition, intestinal bacteria produce vitamins, stimulate the immune system, communicate with the intestinal epithelium and modulate the host’s behavior [2,3]. Communication between the host and microbiome is two-way. On the one hand, microorganisms communicate with the intestinal cells by detecting the host’s hormones and peptides, such as catecholamines. In this way, the microorganisms make sure that they are present in the right place and increase the expression of supporting colonization genes [4]. On the other hand, the microbiome produces a wide range of signaling molecules. Signals from microbial metabolites influence immune maturation, immune homeostasis, host energy metabolism and the maintenance of mucosal integrity. Specific classes of metabolites, notably short chain fatty acids and tryptophan metabolites, have been implicated in the pathogenesis of IBD [5]. The intestinal microbiome is easily altered due to a change of diet or illness. There are also salient differences between individual hosts. Among the microorganisms living in the digestive tract, there are those with positive and negative effects on humans. The beneficial effects of microorganisms are very broad. They include preventing the invasion of disease-causing bacteria and synthesizing essential nutrients and vitamins [6,7,8]. The microorganisms with positive effects can be used to improve health. Microorganisms that, after appropriate preparation and administration, have a positive effect on the body are called probiotics. In 2014, the expert panel from the International Scientific Association for Probiotics and Prebiotics (ISAPP), authorship Hill et al., formulated the definition of probiotics as “live microorganisms which when administered in adequate amounts confer a health benefit on the host” [9]. There is scientific evidence that selected probiotic strains play a significant role in the treatment and prevention of diseases such as diarrhea, lactose intolerance, ulcerative colitis (UC), Crohn’s disease (CD), irritable bowel syndrome (IBS), obesity and cancers, as well as in the development of insulin resistance [10,11,12,13,14,15,16].

Crohn’s disease (CD) and ulcerative colitis (UC) are the main types of inflammatory bowel diseases (IBD). UC is usually limited to diffuse mucosal inflammation, with neutrophils predominating in the lamina propria and crypts of the colon. In CD, inflammation can involve any part of the gastrointestinal tract, but the typical regions are the small intestine, especially the terminal ileum, and the colon. Interactions among genetic and environmental factors, impaired immune regulation, gut barrier dysfunction and changes in the intestinal flora are related to the pathogenesis and development of IBD. Currently, there is a lot of talk about the influence of the gut microbiome (GM) on the development and course of IBD [17,18,19]. Bacterial microflora in patients with IBD differ from that observed in healthy people [20]. There is a considerable reduction in microbiome diversity observed in this group of patients, especially anaerobic ones, with decreased numbers of *Bifidobacterium* spp. and *Lactobacillus* spp. and increased *Bacteroides*, *Escherichia* and *Enterococci* spp. [21,22,23,24,25,26]. One of the suspected mechanisms that can cause intestinal inflammation is a loss of tolerance to commensal bacteria in patients with IBD, which may stimulate an upregulate autoimmunological response to the microbiome [27,28]. Prebiotics, mainly dietary fibers, provide appropriate metabolic substrates for bacteria. These polysaccharides metabolized by microbes are the source of short chain fatty acids (SCFAs), including acetate, propionate and butyrate—the most typical products of probiotics metabolism, called postbiotics. Paraprobiotics, also known as “non-viable probiotics”, “inactivate probiotics” or “ghost probiotics”, refers to both non-viable microbial cells and their fragments. Paraprobiotics are better than probiotics in some clinical cases [29]. This study summarizes the recent data regarding prebiotics, probiotics, synbiotics, paraprobiotics and postbiotics in IBD, classifies clinical trials and research and also shows a possibility for future development.

## 2. Prebiotics

Prebiotics are non-digestible food ingredients which selectively stimulate the growth and the activity of bacterial species in the intestine that have a positive influence on the health of the host organism [30]. The most common prebiotics are inulin, glucooligosaccharides (GOS), fructooligosaccharides (FOS), lactulose and derivatives of galactose and β-glucans [31,32]. Prebiotics are not digested by endogenous enzymes in the human gastrointestinal tract, which is why they reach the colon, and there, they are fermented by bacterial microflora [32,33]. Prebiotics are naturally found in over 36,000 products of plant origin, including artichokes, asparagus, chicory, garlic, onions, wheat and bananas. Additionally, prebiotics may also be artificially produced and introduced into food to increase its nutritional and health value. Prebiotics enhance the number of beneficial bacteria in the human intestine, such as the *Lactobacillus*, *Bifidobacterium* and *Bacteroides* families [33,34]. In order for a product (food or supplement) to be considered a prebiotic, it must meet the following conditions [32]:

- Stimulate the growth and activity of selected strains of bacteria that have a beneficial effect on health.
- Decrease the pH of the intestinal contents.
- To be resistant to hydrolysis and the action of gastrointestinal enzymes.
- To not be absorbed in the upper gastrointestinal tract.
- To provide a medium for one or more beneficial microorganisms in the colon.
- To be stable in the food processing process.

Prebiotics reach the large intestine unchanged and are fermented by the bacteria inhabiting this part of the digestive tract. As prebiotics pass through the lumen of the intestine, they bind to water and increase the volume of the intestinal contents. Due to the loose structure and large surface area, these contents provide a good breeding ground for bacteria. In the process of fermentation of prebiotics, short chain fatty acids are formed, which play an extremely important role in the proper functioning of the intestines. They are a breeding ground for beneficial bacteria and at the same time inhibit the growth of pathogens; accelerate the healing and regeneration processes of the intestinal epithelium; increase the production of mucus; maintain the correct pH in the intestine, which reduces the growth of pathogenic bacteria; increases the absorption of calcium, iron, and magnesium, lowering blood cholesterol levels; and also favorably affects glucose and protein metabolism in the liver [32,33,34,35].

### Recent Clinical Trials on Prebiotics in IBD

Research studies have shown that the consumption of prebiotics greatly affects the composition of the intestinal microbiome and its metabolic activity. This happens due to the modulation of lipid metabolism, increased calcium bioavailability, effects on the system immune function and the modification of intestinal function [36]. However, there are only a few studies regarding prebiotics in IBD published up to now.

Benjamin et al., in their randomized, double-blind and placebo-controlled study on 54 subjects in an intervention group and 49 controls, assessed the impact of FOS on active CD. Patients with active CD were randomized to receive 15 g of FOS or placebo per day for 4 weeks. They found that there was a worsening of the clinical status of patients in the acute stage of CD treated with FOS. Concentrations of probiotic *Bifidobacteria* spp. and *Faecalibacterium prausnitzii* in stool showed no difference between CD and control groups [37]. In another study, performed by Hafer et al., the authors assessed lactulose influence on clinical, laboratory, endoscopic and histopathological activity as well as on quality of life (QoL) in active IBD patients. Authors enrolled 14 active UC patients and 17 active CD patients to the study who were treated with standard therapy alone or combined with 10 g of lactulose daily for 4 months. They found that oral lactulose had no positive effect in active IBD, both UC and CD, on clinical, endoscopic or histopathological activity. However, they observed a significant improvement in QoL in UC patients treated with lactulose compared to controls [38]. Kanauchi et al. performed the open-label multicenter clinical trial with germinated barley foodstuff (GBF) treatment in patients with UC. They enrolled 21 patients with mild-to-moderate active UC and administered GBF for 24 weeks outside of standard therapy with aminosalicylates and/or steroids. After 24 weeks of treatment with the prebiotic, the GBF group showed significant decreases in clinical activity, especially with the presence of blood in the stools and nocturnal diarrhea, compared to the group without GBF treatment [39]. Another study was published by Casellas et al. In their prospective, randomized, placebo-controlled study, they assessed the efficacy of supplementation of inulin enriched with FOS in patients with mild-to-moderate acute UC for 2 weeks. There were ten patients enrolled in the intervention group and nine patients in the placebo group. After 7 days of therapy, a significant decrease in stool calprotectin in the intervention group was observed [40]. Hallert et al., in their randomized, placebo-controlled study, evaluated the efficiency of ispaghula husk supplementation in patients with inactive UC. Twenty-nine patients enrolled in the study with inactive UC were treated with ispaghula husk for 4 months. Afterwards, the intervention group showed a significantly higher clinical improvement rate (69%) compared to the placebo group (24%) [41]. Fernandez-Benares et al., in their study on 105 patients with inactive UC, evaluated Plantago ovata seeds in three groups of patients treated with mesalamine alone, Plantago ovata seeds with mesalamine and Plantago ovata seeds alone for 12 months. They found similar remission rates in all groups of patients. Additionally, a significant increase in stool butyrate levels was observed in the Plantago ovada seeds groups [42]. Hanai et al., in their study, assessed germinated barley foodstuff (GBF) treatment in 59 inactive UC patients for 12 months. He reported significantly lower relapse rates in the GBF group compared to the group without GBF treatment after 12 months of therapy [43]. These data are summarized in Table 1.

The results of prebiotics studies are contradictory, but the main conclusion is that they have no significant positive effect in patients with IBD. However, to date, published controlled trials have been small. The administration of probiotics in acute stages of IBD may also be connected with some gastrointestinal side effects, but it should also be considered that the administration of prebiotics, especially in early childhood, may be crucial for microbiome composition and preventing children from developing IBD in later life.

## 3. Probiotics

Probiotics are live microorganisms that have a positive influence on the gut due to modulating immune response, increasing the production of mucosal IgA and competing with pathological bacteria [44,45]. There are 10 to 100 trillion microbes in the human intestine, and they are commensal organisms that play an important role in humans—for instance, vitamin B synthesis and the digestion process [46,47]—but a modern lifestyle makes it difficult to maintain a healthy gut flora due to stress, hygiene and antibiotic use causing dysbiosis [48]. The biggest group of intestinal bacteria is lactic acid-producing bacteria (LAB), which produce lactic acid during the anaerobic digestion of saccharides. *Lactobacillus* spp. are a main group of bacteria in fermented food such as pickles, soured milk and kefir and are considered to be safe for humans [49]. Due to the development of refrigerated food storage, the need for food preservation by fermentation is diminished, as its consumption could influence the gut microbiota and even cause dysbiosis [48].

Probiotics as live organisms should be able to reach the intestine and help maintain homeostasis. Their most important mechanisms of action are dependent on the strain and include the production of components with antibacterial activity, such as lactic acid, hydroperoxides and bacteriocins; blocking binding sites on epithelial cells; the upregulation of tight junction molecules in mucosal barrier; the degradation of toxin receptors; the modification of pH and competition for essential nutrients [23,24,50,51].

One of the greatest benefits of probiotics is modulating immunity and supporting the immune system’s defenses. In patients with inflammatory bowel disease (IBD), chronic inflammation of the intestine is caused by abnormal activation of the immune system, but with strong participation of genetic and environmental factors, including pathogens [52]. Dendritic cells in the intestinal mucosa are involved in the regulation of the immune system and influence the differentiation of Treg and Th17 as well as the production and switching of IgA classes. Their activation may lead to pro-inflammatory cytokine production (IL-17, IL-23) [53]. Probiotics influence the immune system in the mucosa layer of the intestine and stimulate antibodies’ production by activating Toll-like receptors and T helper 1 differentiation. Deregulation of the immune system reduces the Th17 population and affects the Th17/Treg balance. Probiotics also promote phagocytosis and NK activity, induce T cell apoptosis, stimulate the production of anti-inflammatory cytokines (IL-10, TGF-β) and reduce pro-inflammatory cytokines (TGF-α, If-γ) [36,54,55].

### 3.1. Recent Clinical Trials on Probiotics in IBD

Several clinical trials have evaluated the effectiveness of probiotics in both remission induction and maintenance of IBD, as well as distinguishing between Crohn’s disease (CD) and ulcerative colitis (UC). Selected randomized controlled trials (RCTs) published in recent years are presented below. Tamaki et al. [44] published a randomized and double-blind study performed on 56 patients with mild or moderate UC. Twenty-eight patients received *Bifidobacterium longum* 536, and twenty-eight were in the placebo group. After 8 weeks of observation, significant decreases in clinical activity of the disease, assessed by Ulcerative Colitis Disease Activity Index (UCDAI) score, were observed in the study group (*p* < 0.01) and not in the control group (*p* = 0.88), but the difference between groups was not significant (*p* = 0.5). However, clinical improvement in rectal bleeding was observed. In an endoscopic examination after 8 weeks of treatment, a reduction in endoscopic activity assessed by Mayo scale in the study group was also observed (*p* < 0.01), with no statistically significant reduction in the placebo group (*p* = 0.078). A clinical report presented by Yoshimatsu et al. described the influence of probiotic therapy for preventing relapse of UC in patients in clinical remission. Sixty patients 13 years or older were included in a single-center, randomized, double-blind, placebo-controlled study and divided into two even groups—30 of them were treated with Bio-Three (containing *Streptococcus faecalis T-110*, *Clostridium butyricum TO-A* and *Bacillus mesentericus TO-A*), and the rest received placebo. The observation was performed for one year, and the relapse rate was lower in probiotic group after 3 (0.0% vs. 17.4%), 6 (8.7% vs. 26.1%) and 9 months (21.7% vs. 34.8%), but results were statistically significant only after 3 months (*p* = 0.036). After 12 months of treatment, 69.5% of patients in the Bio-Three group and 56.6% in the placebo group were still in remission, but this difference was not significant (*p* = 0.248) [56]. Another study based on fermented milk was an open-label randomized control, single-center, prospective study performed by Yilmaz et al. on a group of 45 patients with IBD. The patients were divided into two groups, with 24 consuming kefirs with *Lactobacillus* spp. and 20 controls. The study group was administered 400 mL of kefir per day for 4 weeks. After this time, significant decreases in ESR and CRP and higher increases in hemoglobin level in patients with CD using kefir than in controls (*p* = 0.024 vs. *p* = 0.029) were observed. Additionally, levels of bloating (*p* = 0.012) and a subjective feeling good score (*p* = 0.032) were significantly improved in the last two weeks in patients with CD, and the results of the feeling good score (*p* = 0.049) and abdominal pain reduction (*p* = 0.019) were statistically better than in the group with UC in the last two weeks of the study [57]. Another source of probiotic bacteria is yogurt, which was used by Shadnoush et al. Their randomized, placebo-controlled study included 210 patients with IBD and 95 healthy controls. After 8 weeks, they observed increased amounts of *Lactobacillus*, *Bifidobacterium* and *Bacteroides* in the feces of study group participants compared to the control group with IBD (*p* < 0.001, *p* < 0.001, *p* < 0.01) and to the healthy controls (*p* < 0.01, *p* < 0.01, *p* <0.05) [58]. Palumbo et al. published a research study based on a group of patients with moderate-to-severe UC who were treated with mesalazine and the probiotic blend (*Lactobacillus salivarius*, *Lactobacillus acidophilus* and *Bifidobacterium bifidus BGN*4) or mesalazine alone for 24 months. The researchers observed improvement in both groups of patients, but the results in a group treated with probiotics and mesalazine achieved statistically significant improvement compared to the group with mesalazine alone in endoscopic activity as determined by the Mayo Disease Activity Index (*p* < 0.05 after 6, 12, 18 and 24 months), a physician’s global assessment (*p* < 0.05 after 24 months), stool frequency (*p* < 0.05 after 6 and 24 months), endoscopic picture (*p* < 0.05 after 18 and 24 months) and rectal bleeding (*p* < 0.05 after 6, 18 and 24 months). The authors conclude that combined therapy with mesalazine and probiotics could be a good alternative to steroid treatment [59]. Another study comparing drug alone to drug and probiotic was a study conducted by Fan et al. on a group of 40 patients with IBD. Twenty-one of them were treated with mesalazine and Bifico (containing *Enterococcus faecalis*, *Bifidobacterium longum* and *Lactobacillus acidophilus*) and nineteen with mesalazine alone. After 40 days, significant decreases in *Enterobacteria*, *Enterococci*, *Saccharomyces* and *Bacteroides* in stool samples of patients from both groups (to a lower value in the study group) and increases in *Bifidobacteria* and *Lactobacilli* (more significant in study group) (all *p* < 0.05) were observed. The researchers also found differences in inflammatory markers between groups—in the probiotics group, there were significantly lower levels of CRP and IL-6, and higher level of IL-4 than in the mesalazine-only group (all *p* < 0.05). Furthermore, levels of fecal lactoferrin, alpha-1-antitrypsin and beta-2-microglobulin were significantly lower in the study group (all *p* < 0.05). However, the researchers pointed to the small size of the group, short follow-up and low compliance [60]. Su et al. randomized 83 patients with CD to study groups treated with probiotics (*Bifidobacterium, Lactobacillus*) with sulfasalazine and prednisone and treated with sulfasalazine alone. Additionally, 40 healthy, untreated people were recruited to the study as a healthy control group. After treatment, levels of CRP, TNF-α and IL-10 significantly decreased in both CD groups (to a lower value in the probiotic group) and reached the values of the healthy control group. In the study group, better therapeutic efficiency than in the control group was achieved (*p* < 0.05), and the infection rate in the control group was significantly higher (*p* < 0.05) [61]. A study performed by Bjarnason et al. in a group of 81 patients with UC and 61 with CD randomized patients into two groups: multistrain probiotic medicament (Symprove containing *Lactobacillus rhamnosus* NCIMB 30174, *Lactobacillus plantarum* NCIMB 30173, *Lactobacillus acidophilus* NCIMB 30175 *and Enterococcus faecium* NCIMB 30176) and placebo. The study took 4 weeks, and the scientists measured changes in quality of life and laboratory findings. They observed statistically significant improvement only in fecal calprotectin in patients with UC treated with probiotics; the other measured parameters showed no differences between the groups [62]. In 2015, Fedorak et al. performed a multi-center, randomized, placebo-controlled trial on a group of 120 patients with CD who had undergone ileocolonic surgical resection with a small intestine-to-colon anastomosis. Patients were divided into the study group with VLS#3 (containing 900 billion viable bacteria, comprising four strains of *Lactobacillus*, three strains of *Bifidobacterium* and one strain of *Streptococcus salivarius subspecies thermophilus*) and the control group with placebo. During the study, any significant differences in endoscopic signs of recurrence between groups were noticed (9.3% in VLS#3 group vs. 15.7% in placebo group, *p* = 0.19), but the recurrence rate in the placebo group was significantly lower. After one year of observation, patients who started the treatment after surgery had lower rates of severe endoscopic recurrence than the control group, who started VSL#3 from day 91 (10% vs. 26.7%, *p* = 0.09). In addition, significant reductions in mucosal inflammatory cytokine levels were observed in patients receiving probiotics compared to the placebo group (*p* < 0.05) [63]. As fermented milk is a source of *Bifidobacterium breve*, a study performed by Matsuoka et al. examined the effect of the *Bifidobacterium breve* strain *Yakult* (BFM) on relapse-free survival of patients with UC. No differences between groups in relapse-free survival, incidence of relapse or time to worsening were observed [64].

The probiotics studies in IBD are collected in Table 2.

### 3.2. Meta-Analyses of Probiotic Studies in IBD

So far, there have been several meta-analyses made summarizing the current research on probiotics in IBD and giving new quality to the included studies.

A meta-analysis performed by Asto et al. evaluated 18 placebo-controlled studies, published between 1997 and 2018, involving 1491 patients with UC who were treated with probiotics, prebiotics or synbiotics vs. placebo. They did not see any significant effect in maintaining remission in either placebo- or mesalazine-controlled studies, but it is worth noting that they noticed that probiotics could help achieve remission in the active phase of the disease [65]. Another meta-analysis published by Zhang et al. covered 38 studies concerning the effects of not only probiotics (26 studies), but also prebiotics and synbiotics. The results of the analysis showed that probiotics, prebiotics and synbiotics are effective in achieving/maintaining remission, and their use decreased the disease activity index in UC but not in CD. The use of probiotics also increased the population of *Bifidobacteria* in the gut, and the use of synbiotics was of greater benefit than probiotics and prebiotics alone [66]. A meta-analysis published by Jia et al. summarized 10 studies published between 1999 and 2013, most of which (4) concerned *E. coli Nissle* and (3) VSL#3. The analysis showed that there were significant differences between *E. coli Nissle* and mesalazine in the remission, risk of recurrence or occurrence of complications between groups. In the remaining evaluated studies, a statistically significant effect of the intervention on remission and risk of recurrence was demonstrated in the studies with VLS#3, while none of the probiotics showed differences in the occurrence of complications [67]. Puvvada et al. assessed the change in QoL in IBD patients taking probiotics. They analyzed three RCTs—two of them showed a significant improvement in patients’ QoL, and one study found no difference. The researchers concluded that the results of their analysis may indicate that probiotics improve the QoL of patients with IBD [68].

Probiotics are usually administered orally, but this is not the only possible way to apply them. Fecal microflora transplantation is a therapeutic method already used in the treatment of *Clostridium difficile* infection, but it may also be a promising approach in IBD patients. Shen et al. published a paper summarizing reports on the effectiveness of fecal flora transplantation in UC [69]. Most studies were conducted on small groups, and although their results were inconsistent (effectiveness ranged between 20 and 92%), fecal flora transplantation, especially repeated, could be a good therapeutic strategy in IBD patients, but some additional RCT studies on large groups are required [26].

### 3.3. Side Effects of Probiotics Focused on IBD

In 2015, Meini et al. presented the case of a 64-year-old female patient with severe UC, treated with steroids (prednisone), in which the administration of a *Lactobacillus rhamnosus GG* caused bacteremia due to the translocation of bacteria from the intestinal lumen to the blood [70]. In 2019, Dore et al. presented a review of the incidence of side effects in IBD patients treated with probiotics. They evaluated nine trials involving 826 patients. The meta-analysis showed a higher percentage of reported side effects in the group of patients taking probiotics, and this effect was visible in patients with UC—in the group of patients with CD, no significant differences were observed between the groups with the probiotic and the placebo. Patients treated with probiotics more frequently reported gastrointestinal side effects, but statistically significant results were only obtained for the occurrence of abdominal pain [71]. In 2020, the same authors presented a retrospective cohort study on 200 patients with IBD (100 taking probiotics and 100 controls) and assessed their incidence of adverse events related to the underlying disease, such as the need for systemic steroids, hospitalization, and surgery. Most of the patients were administered VSL#3, *Lactobacillus reuteri* (DSM 17938) and a mixture of *S. thermophilus*, *L. acidophilus*, *B. breve* and *B. animalis* ssp. *lactis*. The assessment was performed depending on the disease (UC or CD) and the duration of probiotic use (<24%, 25–75% or >75% disease duration). The best results, lowering the risk of adverse events, were seen in patients taking probiotics during >75% of disease course and were more evident in patients with UC [72].

Although probiotics are considered safe, it must be remembered that in people with reduced immunity, side effects may occur, such as bacterial translocation and sepsis. The benefits of probiotics, however, seem to outweigh the risk of possible side effects in IBD patients, and they may be protective against the side effects of the IBD.

## 4. Synbiotics

Synbiotics are combined probiotics and prebiotics which can show a synergistic beneficial effect on host health [73,74]. Probiotics and prebiotics in combination are considered a promising novel approach, and there is currently an opportunity to evaluate their efficacy and potential use in IBD in humans. However, only a few studies have already been published supporting the use of synbiotic supplementation in IBD. The most frequent synbiotic formulae include *Lactobacillus GG* and/or *Bifidobacteria* with fructooligosaccharides and/or inulin.

Steed et al., in their randomized, double-blind and placebo-controlled study on 35 subjects with active CD in an intervention group, evaluated the influence of synbiotics consisting of *Bifidobacterium longum* and a mix of inulin and FOS. Initially, 35 patients were enrolled to the study, but only 13 patients in the synbiotic group and 11 in the placebo group finished the study and were analyzed. Patients with active CD were randomized to receive 6 g per day of either a synbiotic comprised of a mix of inulin and FOS (Synergy 1) or placebo for 6 months, outside of conventional therapy they received before the enrollment. Clinical response, serum inflammatory markers, cytokine concentrations in mucosal biopsies and probiotic concentrations on the intestinal mucosa were assessed. Authors reported significant improvement in clinical outcomes and decreases in the clinical and histopathological activity of CD, as well as increases of *Bifidobacteria* species in the intestine in synbiotic patients compared to the placebo group, accompanied by a significant decrease in TNF-α in mucosal specimens after 3 months, but not after 6 months [75].

Furrie et al., in a randomized, double-blind and placebo-controlled study, evaluated the same mix of probiotic and prebiotics (Synergy 1) in active UC patients. They enrolled 18 patients with active UC and randomized them to receive a synbiotic including the probiotic *Bifidobacterium longum* and Synergy 1 (mix of inulin and FOS) twice daily for a period of a month. Authors reported no significant differences between clinical activity (*p* = 0.06) but a significant reduction in the endoscopic and histopathological activity in the rectal mucosal specimens. Additionally, the significant decrease in serum CRP and mucosal human beta defensins 2, 3, 4, TNF-α and IL-1 α were found in the synbiotic group compared to the placebo group [76]. Chermesh et al., in a randomized, double-blind and placebo-controlled study, assessed Synbiotic 2000, a synbiotic consisted of four probiotics (*Pediacoccus pentosecens*, *Lactobacillus affinolactis*, *Lactobacillus paracasei susp paracasei 19* and *Lactobacillus plantarum 2362*) and four prebiotics (β-glucan, inulin, pectin and resistant starch). The study included 20 patients in an intervention group treated with Synbiotic 2000 once daily for 24 months and 10 patients in a placebo group. There were no significant differences between the synbiotic and placebo groups in clinical symptoms, laboratory markers or endoscopic activity [77]. Fujimori et al., in their randomized, controlled trial, assessed the efficacy of the probiotic, prebiotic and synbiotic treatments in improving the QoL in patients with UC. There were 120 patients with active and inactive UC enrolled to the study. They were randomized into three groups, the first one treated with probiotic *Bifidobacterium longum*, the second one with prebiotic psyllium and the third one with synbiotic consisted of *B. longum* and psyllium for 4 weeks. At the end of the study, the authors observed significant improvement in the QoL in the synbiotic group compared to the probiotic and prebiotic groups. Additionally, serum CRP significantly decreased in the synbiotic group [78]. Another study published by Ishikawa et al. assessed the efficacy of *Bifidobacterium breve* strain *Yakult* and GOS treatment in UC patients. There were 41 patients with mild-to-moderate UC enrolled to the study, then they were divided to two groups, one with standard therapy and synbiotic treatment for one year and one with standard therapy alone. The results of the study showed a decrease in clinical and endoscopic activity after one year of the synbiotic treatment compared to the non-synbiotic group. Moreover, the amount of myeloperoxidase in the rectal lavage assessed after the treatment also decreased compared to the baseline amount in the synbiotic group [79]. Studies evaluating synbiotics in IBD patients are summarized in Table 3.

However, the approach of investigating the effects of synbiotics on the pathogenic mechanisms of gut inflammation, perhaps to find a potential treatment for IBD and other gut-related disorders, is a new area of research [80]. Therefore, more human and animal studies are needed to collect convincing data and provide a better understanding of their direct effects on health, particularly in IBD.

## 5. Paraprobiotics

The use of probiotics can be dangerous for some people, for example those with reduced immunity, an impaired intestinal barrier, sepsis and premature babies [81,82]. Hence, the concept of administering inactivated bacterial strains, bacterial fragments and products of their metabolism to obtain similar therapeutic effects is highly useful [83,84]. The dead probiotic microbial cells and cell constituents are presently called paraprobiotics. The term paraprobiotics was first used by Taverntiti et al. in 2011 [85].

Paraprobiotics are non-viable and thus easier to store and manufacture, and their mechanism of action is more predictable than probiotics [86,87]. The commercial production process consists of cultivating selected strains of microorganisms and then inactivating them. In the process of their inactivation, methods such as ionizing radiation, ultraviolet rays, high pressure drying and pH changes are used [87]. The subsequent production steps include cleaning processes, e.g., by centrifugation, extraction, and column purification. Their advantages also include the lack of risk of bacterial translocation, a lack of risk of transferring antibiotic resistance genes and being easier to produce, transport and store [88]. Due to the lack of bacterial multiplication, paraprobiotics are administered in strictly adequate amounts, so their therapeutic effects are more precise and reproducible. For all these reasons, paraprobiotics were successfully used even in preterm neonates’ treatment, in which the immune system was compromised [89]. Among the thousands of microorganisms that make up the intestinal microbiome, a dozen or so strains of particular health-promoting importance have been selected and used as paraprobiotics, namely *Bifidobacterium lactis* Bb12, *Bifidobacterium longum*, *Lactobacillus gasseri* OLL2716, *Saccharomyces cerevisiae*, *Lactobacillus brevis* SBC8803 and *Lactobacillus delbrueckii* subsp. *bulgaricus* OLL1073R-1 [86]. The action of paraprobiotics is multidirectional, but the most important of them is their immunomodulation [90]. Proteins and peptides; polysaccharides, including glucans; and fragments of genetic material in the form of AT DNA (e.g., derived from *Lactobacillus* spp.) have such an effect. For instance, proteins and peptidoglycans of lactic acid bacteria and lipoteichoic acid from Gram-positive bacteria can stimulate the immune system or inhibit the excessive response of monocytes [91]. There are no direct studies in IBD patients, but several in vitro studies showed potentially beneficial immunomodulatory effects of paraprobiotics in this group of patients. Fang et al. [92] demonstrated a reduction in the expression of monocyte chemoattractant protein 1 (MCP-1) (*p* < 0.05) and regulation of the expression of TNF-α and IL-12 (*p* < 0.05) in an in vitro model of intestinal mucositis using Caco-2 cells and inactivated *L. rhamnosus*. Additionally, Lopez et al. [93] showed anti-inflammatory properties in Caco-2 cells by ultraviolet-inactivated *Lactobacillus rhamnosus GG* (LGG). UV-inactivated LGG decreased IL-8 production induced with flagellin (*p* < 0.05), and flagellin induced NFκB nuclear translocation, so stimulation of this pro-inflammatory cytokine synthesis was diminished [94]. In addition, some of the paraprobiotic proteins can help regenerate the mucosa and intestinal walls [95], and yeast cell wall components such as β-(1,3)-D-glucan, β-(1,6)-D-glucan, chitin and mannoproteins improve digestion [96,97,98,99,100].

In summary, the use of paraprobiotics, for which there is no risk of inducing side effects related to compromised immunity, may be a good alternative for IBD patients, combining the benefits of probiotics and eliminating the possibility of adverse events. Paraprobiotics have been proven to have anti-inflammatory, immunomodulatory, anti-proliferative and antioxidant properties in in vitro studies which seem to be very promising in preventing and alleviating the symptoms of IBD in humans.

## 6. Postbiotics

The term postbiotics, sometimes also not-quite-correctly called metabiotics, has been recently limited to metabolites/CFS (cell-free supernatants) and soluble factors (products or metabolic byproducts) secreted by live bacteria [89]. The term metabiotics refers to the structural components of probiotic microorganisms and/or their metabolites and/or signaling molecules with a determined chemical structure that can optimize host-specific physiological functions and regulatory, metabolic and/or behavior reactions connected with the activity of host indigenous microbiota [101,102]. They are found in any fermented food, e.g., kefir, kimchi, sauerkraut, tempeh, yogurt and certain pickles, as well as inside the human body. The most important postbiotics are organic acids, short chain fatty acids (SCFA), tryptophan (Trp) and bacteriocins. The benefit of using postbiotics may be direct or indirect [102]. Direct benefits result from the action of postbiotics on the host cells. Indirect benefits are the promotion of the expansion of microbial strains considered beneficial to health and the inhibition of the development of negative strains. The mechanisms of action are discussed later in the article. Depending on the type of microorganism, the strain and the metabolism product, the effects of postbiotics are very different, as is the case with prebiotics. The most important beneficial effects of postbiotics, SCFA in particular, are their anti-inflammatory and antioxidant properties [103].

### 6.1. SCFA

SCFAs are produced by the fermentation of non-starch polysaccharides (NSP), dietary fiber and resistant starch in the human intestine [104,105]. A particularly important role is assigned to dietary fiber [106]. The composition of SCFA depends on the substrates and the microorganisms digesting these substrates. The resulting products have two to six carbon atoms in the aliphatic chain [103]. There are three main SCFAs: acetic acid (AA), propionic acid (PA) and butyric acid (BA). Those SCFA are produced in almost all parts of the intestine, but mainly in the proximal part of the large intestine [103]. The proportions between SCFAs, as well as the total concentration of SCFA, are not constant. They depend on the diet, age and disease of the patient [107]. The optimal acetate, propionate and butyrate ratio in the large intestine is 60:25:15, and the SCFA total concentration is between 70 and 140 mM [108]. A correct SCFA ratio helps maintain immune system homeostasis. The interaction of SCFA with cells of the immune system takes place through the free fatty acid receptors (FFAR), a group of G protein-coupled receptors presented on the cell membrane. The majority (95%) of SCFAs are absorbed into the portal vein and transported to the liver, where they are further transformed or degraded. Less than 5% of SCFAs are excreted with the feces [109]. SCFAs as organic acids have the potential to acidify the environment. Some researchers claim that it is beneficial, having improved the bioavailability of metals and provided a protective barrier against the colonization of pathogenic microorganisms [110,111]. However, others suppose that the acidification of intestinal contents may damage the intestinal barrier [112].

#### 6.1.1. Butyric Acid (BA)

BA is one of the most potent SCFAs despite making up a relatively low percentage of SCFA (around 15%). In humans, BA acts in two ways: intestinal and parenteral. The intestinal effects of BA are multidirectional. BA serves as one of the primary energy sources for colonocytes and has a protective effect by increasing the expression of mucin genes such as MUC2 and, consequently, increasing mucin production [113]. BA has a stimulating effect on the proliferation of enterocytes and inhibits the growth of colon cancer cells. This effect is called the “butyrate paradox”. It results from the inhbition of histone deacetylase (HDAC) [114]. Butyrate inhibits the activity of the NF-κB complex in cells of the immune system. In this way, the expression of genes responsible for the synthesis of the major pro-inflammatory cytokines TNFα, IL-1β, IL-2, IL-6, IL-8 and IL-12 is reduced. Some important qualities of BA are its antioxidant potential and its ability to increase the amount of reduced glutathione [115]. Attempts have been made to support IBD treatment with rectal infusions of butyric acid or to administer it orally in the form of various preparations [116,117,118]. However, the results of the studies are inconclusive. Doubts exist with respect to the effects of treatment, the sample size and the poor availability of preparations with a standardized release of butyric acid [119]. The parenteral effect of BA is also based on the inhibition of HDAC and contributes to the improvement of anemia, common in IBD, by promoting hemoglobin synthesis and increasing the number of reticulocytes [120]. Most butyrate is metabolized by the colonic epithelium, resulting in low levels of butyrate in the portal vein, ranging in humans from 1.3 to 14.4 µM [121,122,123,124]. BA binds specifically to the GPR109A receptor. The receptor is expressed in adipocytes and cells of the immune system (dendritic cells, macrophages, monocytes, neutrophils) [125]. Activation of the receptor causes the secretion of anti-inflammatory IL-10 and aldehyde dehydrogenase (ALDH1). It also supports the detoxification process and removal of electrophilic compounds in addition to enhancing Treg lymphocyte differentiation [126].

#### 6.1.2. Propionic Acid (PA)

Propionic acid is comprised of three carbonic acids and occurs naturally in milk and other dairy products. It is produced in the process of natural fermentation by *Propionibacterium*. It is also added as a food preservative. It has antibacterial and antifungal properties. However, PA delivered with the diet is a small amount as compared to PA produced in the intestine [127]. In the colon, PA is produced by the fermentation of polysaccharides and oligosaccharides and the degradation of long chain fatty acids, proteins, peptides and glycoproteins by the anaerobic microbiota [104]. In contrast to BA, most of the PA produced in the intestines is absorbed and transported through the portal vein. Then, about 90% of the absorbed PA is metabolized by the liver [128]. The protective action of PA is based on preventing colonization of the intestinal lumen by pathogenic bacteria from the *Salmonella* family. It is carried out by inhibiting the genes of invasive bacteria [129]. PA also inhibits the activity of COX enzymes by inhibiting the formation of prostaglandins or prostacyclin and inhibiting the development of local inflammation [130]. The anti-inflammatory properties of PA are seen at high concentrations, above 3 mM, which may be present in the intestine. At such concentrations, PA inhibits lymphocyte proliferation and activates the secretion of anti-inflammatory resistin in adipose tissue. In addition, a high concentration of PA inhibits LPS-stimulated TNF-α release by human neutrophils and endothelial cells [131]. PA is the most potent ligand of GPCR43. This receptor is strongly exposed to cells of the immune system, which proves a strong relationship between the PA produced by bacteria and the immune system [132].

#### 6.1.3. Acetic Acid (AA)

There is also a lot of controversy around AA. Elevated concentrations of AA may promote the growth of neoplastic tissue. During hypoxia, AA is an epigenetic modulator that promotes lipid synthesis in neoplastic cells [133] and thus blunts tumor proliferation. The mechanism of this reaction is not fully understood, but it is related to histone h3 hyperacetylation in hypoxic cells. Another study showed that elevated AA levels promote the onset of metabolic syndrome. It has been proven in an animal model that activation of the parasympathetic system by an excessive amount of AA produced by the intestinal microbiome causes an increase in insulin and ghrelin secretion and, consequently, hyperphagia and obesity [134]. AA functions based on interaction with the GPR43 receptor [135,136].

### 6.2. SCFAs in Treatment of IBD

SCFAs are considered a promising adjuvant therapy in the current clinical management of patients with active IBD. Various approaches have been used, including butyrate enemas and combinations of different SCFAs.

In 2002, Lührs et al., in their study, compared the effectiveness of a butyrate enema versus a placebo. Their patients used enemas twice daily for 8 weeks. The enemas consisted of 60 mL of 100-mM sodium butyrate. Eleven people with UC were qualified for the study. Six patients received butyrate enemas and five received placebo. Patients treated with sodium butyrate displayed a significant reduction in the number of macrophages with the expression of NF-kB. At first, a colonoscopy was performed, and mucosal biopsies were obtained. Follow-up examinations after 4 and 8 weeks included a rectosigmoidoscopy. In this study, two biopsies were taken from the rectum (10 cm from the anal verge) and another two biopsies from the rectosigmoid junction (20 cm from the anal verge). In addition, butyrate significantly reduced the number of neutrophils in crypt and surface epithelia and the lamina propria lymphocytes/plasma cells. The results correlated with a decline in the Disease Activity Index (DAI). In patients not treated with butyrate, NF-kB was observed in all macrophages [113]. Senagore et al. in 1992 compared the effectiveness of corticosteroid, mesalazine and SCFA enemas in patients with proctosigmoiditis (in the course of UC). They received SCFA mixed in a ratio of 46% acetate to 23% propionate to 31% butyrate in enemas twice daily for 6 weeks. The total concentration was 130 mM, with a volume of 60 mL. This study performed a randomized, prospective comparison of corticosteroid (enemas of 100 mg of hydrocortisone, *n* = 12) and mesalazine enemas (4 g of mesalazine, *n* = 19) with SCFA (*n* = 14). Recovery occurred in a similar proportion in patients of these three groups. This study indicates that SCFA enemas are equally efficacious as CS or mesalazine enemas for the treatment of UC [137]. Vernia et al., in their research from 1995, used enemas twice daily for 6 weeks with a mix of SCFA in the proportion of 53% sodium acetate, 20% sodium propionate, 27% sodium butyrate. The total concentration was 150 mM, with a volume of 100 mL. Nineteen patients participated in the study, fourteen were treated with SCFA and five received a placebo. In the SCFA-treated group, health benefits such as decreases in intestinal bleeding (*p* < 0.05) and urgency (*p* < 0.02), as well as a raised patient self-evaluation score (*p* < 0.05) compared to placebo-treated group, were observed. No group displayed a difference in the number of bowel motions [138]. In 2010, Hamer et al. used 60 mL of rectal enemas containing 100-mM sodium butyrate (*n* = 17) or saline (*n* = 18) for 20 days in patients with distal UC in clinical remission. Butyrate enemas induced minor effects regarding colonic inflammation and oxidative stress. Only a significant increase of the colonic IL-10/IL-12 ratio was found within butyrate-treated patients (*p* = 0.02), and colonic concentrations of CCL5 were increased after butyrate compared to placebo treatment (*p* = 0.03). Furthermore, the effect of butyrate on colonic glutathione levels has been proven, but in general, butyrate did not affect glutathione levels in colonic biopsies [115]. In 1992, Scheppach et al. enrolled 10 patients with distal UC treated with oral corticosteroids or mesalazine in whom conventional therapy was unsuccessful for 8 weeks. They were treated for 2 weeks with sodium butyrate (100 mM) enemas and 2 weeks with placebo in a random order (single-blind trial). After butyrate irrigation, stool frequency (*n*/day) decreased from 4.7 +/− 0.5 to 2.1 +/− 0.4 (*p* < 0.01), and discharge of blood ceased in 9 of 10 patients. The authors developed a scale against which they assessed the endoscopic and histological results. The endoscopic examination was based on the appearance of the intestinal mucosa, while the histological examination was based on H&E staining for inflammatory changes. The endoscopic score fell from 6.5 +/− 0.4 to 3.8 +/− 0.8 (*p* < 0.01). The histological degree of inflammation decreased from 2.4 +/− 0.3 to 1.5 +/− 0.3 (*p* < 0.02). There were no such changes in the placebo group [139]. Scheppach et al., also in 1996, conducted another study on the effects of SCFA on IBD. They administered 60-mL enemas of 130-mM SCFA (46% acetate, 23% propionate, 31% butyrate), 100-mM butyrate or saline placebo to 47 patients with active distal ulcerative colitis twice daily for 8 weeks. The effects were assessed immediately and after 4 and 8 weeks of therapy. The DAI was decreased but not statistically significant. The endoscopic appearance of the mucosa and the histologic degree of inflammation were not different among the groups. After eight weeks, fewer colonic segments were affected endoscopically following butyrate treatment than placebo. This study showed trends towards a beneficial effect of SCFA [140]. Steinhart, in 1996, examined 38 patients with distal ulcerative colitis who were randomly assigned to receive nightly 60-mL enemas of butyrate at a concentration of 80 mM (*n* = 19) or placebo (*n* = 19) for 6 weeks. Patients were assessed clinically and endoscopically at baseline and 3- and 6-week follow-ups. Pre- and posttreatment mucosal biopsies were assessed histologically. Clinical improvement was seen in seven subjects treated with butyrate and nine subjects treated with placebo. The efficacy of treatment with butyrate enemas was not proven [141]. Additionally, in 1997, Breuer et al. did not notice any improvement after using SCFA enemas. In their study, 103 patients with distal ulcerative colitis were entered into a six-week, double-blind, placebo-controlled trial using 100 mL of 150-mM SCFA enemas (53% acetate, 20% propionate, 27% butyrate) twice daily for 6 weeks. Of the 91 patients completing the trial, 33% of patients in the SCFA-treated and 20% in the placebo-treated group improved during the study. The difference suggests a trend but was not statistically significant. Those treated with SCFA also had better, but also statistically insignificant, reductions in every component of their clinical and histological activity scores. Only patients with colitis dating back less than 6 months responded more to SCFA than to placebo (48% vs. 18%, *p* = 0.03) [142]. The results of SCFA studies are summarized in Table 4.

Most of the research described supplementation of SCFA in active phases of UC. The results are inconsistent; some studies have described the beneficial effect of SCFA on clinical parameters as well as patients’ well-being, while other studies have not found any significant improvement [143].

### 6.3. Tryptophan (Trp)

One of the main elements of the immune balance in the gut is tryptophan and its metabolites [144,145]. Tryptophan is a critical regulator of inflammation involved in the fine-tuning of adaptive immunity, mucosal barrier function and the maintenance of intestinal homeostasis [146]. The metabolism of Trp in the intestine consists of three main pathways: (1) direct metabolism by the intestinal microbiome, (2) kynurenine pathway in the host’s cells and (3) enzymatic transformation to serotonin (5-HT). The intestinal microbiome metabolizes tryptophan, which regulates the amount of tryptophan available to the host, influencing serotonin and the immune system [147]. Many products of the microbial metabolism of tryptophan, such as indole-3-acetic acid, tryptamine, indole-3-aldehyde (I3A), indole-3-acid-acetic (IAA), indole-3-propionic acid (IPA), indole-3-acetaldehyde (IAAl) and andindoleacrylic acid, are ligands for the aryl hydrocarbon receptor (AhR). AhR is a transcription factor that mediates the expression of genes involved in the metabolism of xenobiotics, including dioxins or drugs metabolized by the cytochrome P450 complex. Tryptophan metabolites produced by microorganisms such as AhR ligands are crucial in protecting the mucosa against inflammation. The AhR can directly promote specific genes, including IL-6, IL-22, PTGS2 VEGFA and cytochrome P450 1A1, which is a direct AhR transcription target providing a feedback loop for AhR signaling [148,149]. AhR signaling is considered a key component of the immune response at barrier sites and is thus crucial for intestinal homeostasis by acting on epithelial renewal, barrier integrity and many immune cell types, such as epithelial lymphocytes, Th17 cells, innate lymphoid cells, macrophages, dendritic cells and neutrophils. Metabolism of tryptophan by the intestinal microbiome leads to the production of bioactive postbiotic derivatives such as indole acetate and propionate indole. These compounds have proven anti-inflammatory effects [150]. This suggests that decreased Trp metabolism among gut microbes may adversely affect IBD [151,152]. In an animal model, decreased concentrations of indole acetate and indole propionate have been demonstrated in the course of IBD. Host enzymes involved in tryptophan metabolism have a beneficial effect in relieving IBD symptoms. The IDO1 enzyme is involved in the regulation of acquired immunity. The enzyme catalyzes the conversion of tryptophan by kynurenine. Overexpression of IDO1 is observed locally in the gut and systemically. This hypothesis is confirmed by the higher activity of IDO1 in IBD compared to inactive IBD patients and the negative correlation between the levels of Trp and C-reactive protein [153]. The presence of the enzyme is observed in intestinal mononuclear cells such as T lymphocytes. These cells are present in inflammatory infiltrates located in the intestinal wall [154]. The role of the IDO1 receptor in immunosuppression is multifaceted and includes the suppression of CD8+ T effector cells and NK cells, as well as the increased activity of TregCD4 + regulatory cells [155]. Patients with IBD have elevated levels of tryptophan metabolites (kynurenine and kynurenic acid) and a decreased level of tryptophan in plasma. Similar findings are seen in the GI tract. It is suggested that pro-inflammatory cytokines such as IL-2, IL-6 and TNF-α promote the catalytic conversion of tryptophan to its metabolites. In patients with IBD, increased expression of IDO1 has been observed on peripheral blood lymphocytes and lymphocytes present in the colon [156]. Moreover, these patients have elevated levels of serotonin in the intestinal mucosa. This has been suggested to be of importance in the development of intestinal inflammation [157]. About 1–2% of ingested Trp is metabolized to serotonin. Serotonin affects not only the CNS but also the gastrointestinal tract. Approximately 90% of total serotonin is produced in enterochromatophilic cells. 5-HT is an important gastrointestinal signaling molecule that transmits signals from the gut’s neurons and influences gut motility, vasodilatation, secretion and nutrient absorption [158]. Changes in Trp and its metabolites in the disease suggest that they may be therapeutic targets. For example, the administration of *Lactobacillus*, which naturally produces AhR agonists, alleviates colonic inflammation in mice with genetically induced dysbiosis, suggesting potential therapeutic applications in IBD [159]. Similarly, *Lactobacillus reuteri*, by producing the AhR agonist indole-3-lactic acid, can reprogram intraepithelial CD4+ T cells into CD4+ CD8aa+ immunoregulatory T cells [160]. When IDO1 is over-activated, such as in inflammatory bowel conditions, the reduced availability of Trp may contribute to the lower production of AhR agonists by the gut microbiota. In this situation, Trp supplementation helps to alleviate the symptoms of colitis in mice and pigs with induced inflammation [161,162].

## 7. Conclusions

The majority of described studies have significant limitations, such as small study populations and a lack of analysis with concomitant treatment, including immunosuppressants, steroids and aminosalicylates, which can affect outcome.

Nevertheless, prebiotics have yet to be shown to have any positive effect in IBD, but to date, published controlled trials have been small. The administration of probiotics may pose some risk for the patients and should not be assumed to be innocuous, especially when ingested by patients with an impaired intestinal barrier. Prebiotics may not be harmful but may relate to some gastrointestinal side effects. Finally, the time of administration of prebiotics, especially in early childhood, may be important to shape the bacterial microbiome of the gastrointestinal tract and its predisposition to or prevention of the development of IBD. Finding ways to impact the gut microbiome to alter the course of IBD makes good sense but should be undertaken in the setting of rigorously performed controlled trials to ensure that the interventions are truly effective and well-tolerated.

Research on the use of probiotics in IBD shows inconclusive results. There are indications that selected probiotics may be effective in inducing and maintaining remission, but this effect is more pronounced in UC patients than in CD patients. Among the available probiotics, VLS#3 brings the greatest benefit from supplementation, although not all studies confirm this effect. The use of multi-strain probiotics appears to bring more benefits to patients than the administration of single-strain probiotics. Due to the large number of probiotics whose effects are assessed by clinical trials, it is difficult to compare the results between individual studies [56,57,58,59,60,61,62,63,64]. More research is needed to assess the effects of individual probiotics in IBD patients.

Although synbiotics seem to have more health benefits for the host organism than probiotics or prebiotics alone, it is impossible to draw definitive conclusions because of the variation in their benefits, likely based on the different combinations of the types and doses of prebiotics and probiotics.

Paraprobiotics are non-viable and thus easier to store and manufacture, and their mechanism of action is more predictable than probiotics. Their advantages also include the lack of risk of bacterial translocation, the lack of risk of transferring antibiotic resistance genes, the effect on epithelial cells being more precise and being easier to produce, transport and store. The use of paraprobiotics, for which there is no risk of inducing side effects related to compromised immunity in patients with IBD, may be a good alternative, combining the benefits of probiotics and eliminating the possibility of adverse events, but this should be proven by RCT studies.

Research on postbiotics, especially SCFA, nowadays is not so common. Because studies were not standardized, the results are inconsistent; however, most of them have described the beneficial effect of SCFA. Extensive research is needed to link specific prebiotics to specific probiotics and the resulting postbiotics in IBD patients.

A potential area for future research, besides properly designed studies on paraprobiotics and postbiotics in IBD patients, is the personalized combination of prebiotics and probiotics or paraprobiotics and postbiotics, especially if patient-dedicated (personalized) nutritional intervention in IBD patients is a very important part of modern IBD therapy. Such personalized holistic therapy, biotics in combination with nutritional and pharmacological therapy, will increase the effectiveness of treatment while reducing side effects.

## Figures and Tables

**Table 1:** Studies evaluating prebiotics in IBD patients.

Study	Subjects	Intervention	Number of Patients	Duration of the Study	Outcome
Benjamin JL et al., 2011	Active CD	FOS vs. placebo	54 with CD and 49 controls	4 weeks	Deterioration of clinical status of CD patients; no significant differences in *Bifidobacteria* spp. and *F. prausnitzii* stool concentrations
Hafer A et al., 2007	Active UC and CD	Lactulose	14 with UC and 17 with CD patients	4 months	No significant improvement in clinical, endoscopic and histopathological activity; improvement in QoL in UC patients
Kanauchi O et al., 2003	Active UC	Germinated barley foodstuff	21 with UC	24 weeks	Significantly decrease the clinical activity of the UC in the prebiotic group, especially presence of blood in the stools and nocturnal diarrhea
Casellas F et al., 2007	Active UC	Inulin and FOS	19 with UC	2 weeks	Significant decrease of stool calprotectin after 7 days of treatment in intervention group
Hallert C et al., 1991	Inactive UC	Ispaghula husk	29 with inactive UC	4 months	Significant clinical improvement in intervention group
Fernandez-Benares F et al., 1999	Inactive UC	Plantago ovata seeds	105 patients with inactive UC	12 months	Similar remission rates in groups treated meslamine, mesalamine and Plantago ovada seeds and Plantago ovada seeds alone; significant increase of stool butyrate level in Plantago ovada seeds groups
Hanai H et al., 2004	Inactive UC	Germinated barley foodstuff	59 patients with inactive UC	12 months	Significantly lower relapse rate in GBF group compared to group without GBF treatment

**Table 2:** Studies evaluating probiotics in IBD patients.

Study	Subject	Intervention	Number of Patients	Duration of the Study	Outcome
Tamaki et al., 2016	Remission induction in UC	*Bifidobacterium longum 536* vs. placebo	56	8 weeks	Significant improvement in UCDAI (*p* < 0.01) and MAYO score in study group; no improvement in control group
Yoshimatsu et al., 2015	Inactive UC	Bio-Three vs. placebo	60	12 months	Lower relapse rate in probiotic group after 3, 6 and 9 months (statistical significance only after 3 months); remission rate higher in probiotic group than placebo (69.5% vs. 56.6%, *p* = 0.248)
Yilmaz et al., 2019	IBD	Kefir vs. no intervention	45	4 weeks	Significant decrease in ESR, CRP; increase in hemoglobin, reduced bloating and increase in well-being in study group
Shadnoush et al., 2015	IBD	*Lactobacillus acidofilus*, *Bifidobacterium* vs. placebo	210 patients with IBD, 95 healthy individuals	8 weeks	Significant increase of *Lactobacillus*, *Bifidobacterium* and *Bacterodies* population in study group (*p* < 0.001, *p* < 0.001, *p* < 0.01)
Palumbo et al., 2016	Moderate-to-severe UC	*Lactobacillus salivarius*, *Lactobacillus acidophilus* and *Bifidobacterium bifidus strain* BGN4 with mesalazine vs. mesalazine	60	2 years	Better improvement in study group compared to the control group in recovery time, disease activity and endoscopic picture
Fan et al., 2019	IBD	Probiotic (Bifico) with mesalazine vs. mesalazine alone	40	40 days	Significant decrease in hs-CRP and IL-6, increase of IL-4 and decrease in fecal lactoferin, alfa-1-antitripsin and beta-2-microglobulin in study group compared to control (all *p* < 0.05)
Su et al., 2018	CD	*Bifidobacterium* and *Lactobacillus* with sulfasalazine and prednisone vs. sulfasalazine	83 + 40 healthy individuals	?	Level decrease of CRP, TNF-α and IL-10 in both groups, significantly lower in study group (*p* < 0.05); significantly higher treatment effect in study group (*p* < 0.05); higher infection rate in control group (*p* < 0.05)
Bjarnason et al., 2019	CD and UC	Multi strain probiotic (Symprove) vs. placebo	81 with UC and 61 with CD	4 weeks	No significant differences in IBD QoL; no significant changes in laboratory tests; statistically significant improvement in fecal calprotectin level in UC, but not in CD
Fedorak et al., 2015	CD after ileocolonic surgical resection with a small intestine to colon anastomosis	VSL#3 vs. placebo	120 (58 in a study group, 62 in a control group)	90 days and 365 days	No significant differences between groups after 90 days; in one-year observation, lower incidence of severe endoscopic recurrence in a group with VLS#3 from post-resection (*p* = 0.09); reduction in inflammatory cytokine levels in probiotic group after 90 days (*p* < 0.05)
Matsuoka et al., 2018	Maintaining remission in UC	BFM fermented milk vs. placebo	195	48 weeks	No significant differences between groups; study discontinued

**Table 3:** Studies on synbiotics in IBD.

Study	Subjects	Intervention	Number of Patients	Duration of the Study	Outcome
Steed H et al., 2009	Active CD	*Bifidobacterium longum* and a mix of inulin and FOS vs. placebo (Synergy 1)	35 with CD	6 months	Significant decrease of clinical and histological activity of CD; significant reduction of TNF-α in mucosal specimens after 3 months
Furrie E et al., 2005	Active UC	*Bifidobacterium longum* and a mix of inulin and FOS vs. placebo (Synergy 1)	18 with UC	1 month	No significant differences between clinical activity, but significant reduction of endoscopic and histopathological activity in the mucosal specimens, accompanied by decrease of hBD 2, 3, 4, TNF-α and IL-1 α
Chermesh I et al., 2006	CD patients after surgical resections	Synbiotic 2000	30 with CD	24 months	No significant improvement in clinical, laboratory or endoscopic activity
Fujimori S et al., 2009	Active and inactive UC	*Bifidobacterium longum* and *psyllium* alone and their combination as synbiotic	120 with UC	4 weeks	QoL significantly increased in synbiotic group compared to probiotic and prebiotic groups; CRP significantly decreased in synbiotic group
Ishikawa H et al., 2011	Maintaining remission in UC	*Bifidobacterium breve* strain *Yakult* and GOS	41 with active UC	12 months	Significant reduction of clinical and endoscopic UC activity in synbiotic group; decrease of myeloperoxidase amount in rectal lavages as disease activity

**Table 4:** SCFAs in the treatment of IBD.

Author/Year	Study	Clinical Group	Relevance
Lührs H. et al., 2002	60 mL of 100-mM sodium butyrate enemas twice daily for 8 weeks	6 with UC	Butyrate treatment for 4 and 8 weeks resulted in a significant reduction in the number of macrophages being positive for nuclear translocated NF-kappaB.
Senagore AJ et al., 1992	60 mL of 130-mM SCFA (46% acetate, 23% propionate, 31% butyrate) enemas twice daily for 6 weeks	40 with UC	SCFAs equally efficacious to corticosteroids or 5-aminosalicylic acid
Vernia P. et al., 1995	100 mL of 150-mM SCFA (53% acetic, 20% propionate, 27% butyrate) enemas twice daily for 6 weeks	14 with UC	Low intestinal bleeding, urgency, rise patient self evaluation score
Hamer HM. et al., 2010	60 mL of 100-mM sodium butyrate enemas for 20 days	17 with UC	A significant increase in the colonic IL-10/IL-12 ratio was found within butyrate-treated patients
Scheppach W. et al., 1992	100 mL of 100-mM butyrate enemas twice daily for 2 weeks	10 with UC	Low stool frequency and endoscopic and histological scores
Scheppach W. et al., 1996	60 mL of 130-mM SCFA (46% acetate, 23% propionate, 31% butyrate) vs. 100-mM butyrate vs. placebo 60 mL twice daily for 4–8 weeks	47 with UC	No differences between groups.
Steinhart AH 1996	60 mL of 80-mM butyrate enemas for 6 weeks	38 with UC	No differences in the study group
Breuer RI 1997	100 mL of 150-mM SCFA (53% acetate, 20% propionate, 27% butyrate) enemas twice daily for 6 weeks	103 with UC	No therapeutic value
